# *Mycobacterium w - a* promising immunotherapeutic intervention for diseases

**DOI:** 10.3389/fimmu.2024.1450118

**Published:** 2024-10-29

**Authors:** Kirsten Stefan, Ryan Gordon, Annah Rolig, Alexander Honkala, Dhanir Tailor, Lara E. Davis, Rajiv I. Modi, Manjul Joshipura, Bakulesh Khamar, Sanjay V. Malhotra

**Affiliations:** ^1^ Department of Cell, Development & Cancer Biology, Knight Cancer Institute, Oregon Health & Science University, Portland, OR, United States; ^2^ Center for Experimental Therapeutics, Knight Cancer Institute, Oregon Health & Science University, Portland, OR, United States; ^3^ Division of Hematology/Medical Oncology, School of Medicine, Oregon Health & Science University, Portland, OR, United States; ^4^ Research & Development Center, Cadila Pharmaceuticals Ltd, Dholka, Gujarat, India

**Keywords:** immune therapy, *Mycobacterium w*, vaccine, leprosy, cancer, tuberculosis, COVID-19

## Abstract

Immunomodulating agents interact with the immune system and alter the outcome of specific immune processes. As our understanding of the immune system continues to evolve, there is a growing effort to identify agents with immunomodulating applications to use therapeutically to treat various diseases. *Mycobacterium w* (Mw), a heat-killed mycobacterium, is an atypical mycobacterial species that possesses strong immunomodulatory properties. Mw was initially evaluated as an immune-therapeutic against leprosy, but since then Mw has generated a lot of interest and been studied for therapeutic applications across a host of diseases, such as pulmonary tuberculosis, tuberculous pericarditis, sepsis, lung cancer, and more. This article summarizes a large body of work published in the past five decades, describing various aspects of Mw and its potential for further therapeutic development.

## 1 Introduction

Immunomodulating agents have broad disease applications, spanning bacterial infections all the way to cancer. As our understanding of the immune system continues to evolve, so does our understanding of its complex interplay with a wide spectrum of pathogenic disease processes. This knowledge has resulted in ever increasing efforts to identify new compounds or to repurpose compounds to harness the immune system to improve patient outcomes.

Heat killed *Mycobacterium w* (Mw) is one such immunomodulator. Mw was isolated at the All India Institute of Medical Sciences and was selected from 71 *Mycobacteria* based on its immunomodulatory properties (1). The name originated as a result of coding given to the strains of *Mycobacteria* investigated in the Talwar laboratory (2). However, to avoid confusion with the multidrug-resistant strain W of *Mycobacterium tuberculosis*, a new name for Mw was suggested, *M. indicus pranii* (MIP), based on the site of isolation from India (indicus), the discovery by Dr. Pran Talwar (pran), and characterization at the National Institute of Immunology (3–5). Mw is a cultivable, non-pathogenic, and rapidly growing saprophyte. Mw is classified in Runyons group IV along with other rapid growers like *Mycobacterium fortuitum*, *Mycobacterium smegmatis*, *Mycobacteroides chelonae*, and *Mycobacterium vaccae* based on their growth, metabolic properties, biochemical features, gene profile, chemotaxonomic features, metabolic machinery, Raman spectroscopy, and KEGG pathways (1–3, 6–12). Mw is tolerant to isoniazid, sodium chloride, and carboxylic acid hydrazide (9). As per molecular and proteomic analysis, Mw is placed way above *M. tuberculosis* in evolutionary scale and is predecessor of mycobacterium avium intracellular complex (2, 10).

Mw has been extensively studied for its immunomodulatory properties. For example, Mw administration is associated with antigen specific improvement in T-cell-mediated immunity and efficient enlargement of draining lymph nodes in mice (13). Investigations in guinea pig models indicated Mw induced immunity has strong cross-reactivity with *Mycobacterium leprae* (14). Because of this cross-reactivity, Mw was first deployed as a vaccine to prevent leprosy and then subsequently explored for its potential to prevent tuberculosis (TB). Lepromin testing indicates host resistance to *Mycobacterium leprae*. A positive finding following bacillary suspension injection implies a cell-mediated immune response, while a negative test suggests lack of disease resistance. In early clinical trials for leprosy, Mw was evaluated for its ability to convert healthy contacts of leprosy patients from lepromin negative to lepromin positive, and leprosy patients to lepromin positive (a surrogate for therapeutic efficacy) following intradermal (ID) Mw injection (15–18). In healthy contacts of leprosy patients, conversion was identified in the majority after a single injection, but improved with subsequent injection. The conversion rates from two separate trials were 82.35% (n=68) and 73.6% (n=72) after single injection, which improved to 98.5% (n=68) and 87.5% (n=72) after the second injection (17, 18). In leprosy patients, the lepromin conversion was slightly lower than healthy contacts after single injection 62.5% (n= 32), which improved to 93.1% (n= 32) and 90.2% (n=162) after multiple injections (15, 16). Attempts to identify antigenic fractions of Mw using peripheral blood from patients vaccinated with Mw revealed a 28-31 kDa antigenic fraction that activated T- and B-cell determinants (19). The 18 kDa, 30 kDa, and 65 kDa antigens of Mw share B and T cell determinants with both *M. leprae* and *M. tuberculosis* (20, 21).

These initial positive findings prompted expanded efforts to develop Mw further. When Mw was used as an immunotherapy in conjunction with a standard chemotherapy regimen in patients with multibacillary leprosy, both skin lesions and clinical status improved and bacterial clearance was observed (4, 22). Prophylactic studies performed in parallel with clinical trials of household contacts of leprosy patients found a Mw vaccine to be highly effective in preventing *M. leprae* infection in at risk populations (23). This and other studies eventually led to the approval of a Mw vaccine (manufactured by Cadila Pharmaceuticals) by the Central Drugs Standard Control Organization of India for management of leprosy. Additionally, the insights gained using Mw to treat leprosy opened the door to other, expanded indications. Therefore, in this review, we discuss the mechanism of action of Mw, and the preclinical and clinical experience in using Mw for management of a wide spectrum of diseases including TB, gram negative sepsis, cancer, and COVID-19.

## 2 Mw mechanisms of action

### 2.1 Immune-based mechanisms of action

Early studies indicated that following exposure to the Mw vaccine, T cells in mice predominately upregulated IL-2 and IFN-γ (24). Further, the immune-stimulating effect of Mw was strong enough to enable lymphocytes from leprosy patients to secrete macrophage activating factors (23, 25). The cytokine profile initiated by Mw, suggests immunity was conferred primarily by Th1 cells (24). Further, *in vivo* depletion of CD4^+^ and CD8^+^ T cells in mice impaired the ability of Mw to confer resistance to TB, as assessed by colony formation, cytokine production, and *in vivo* disease (26).

**Figure 1:**
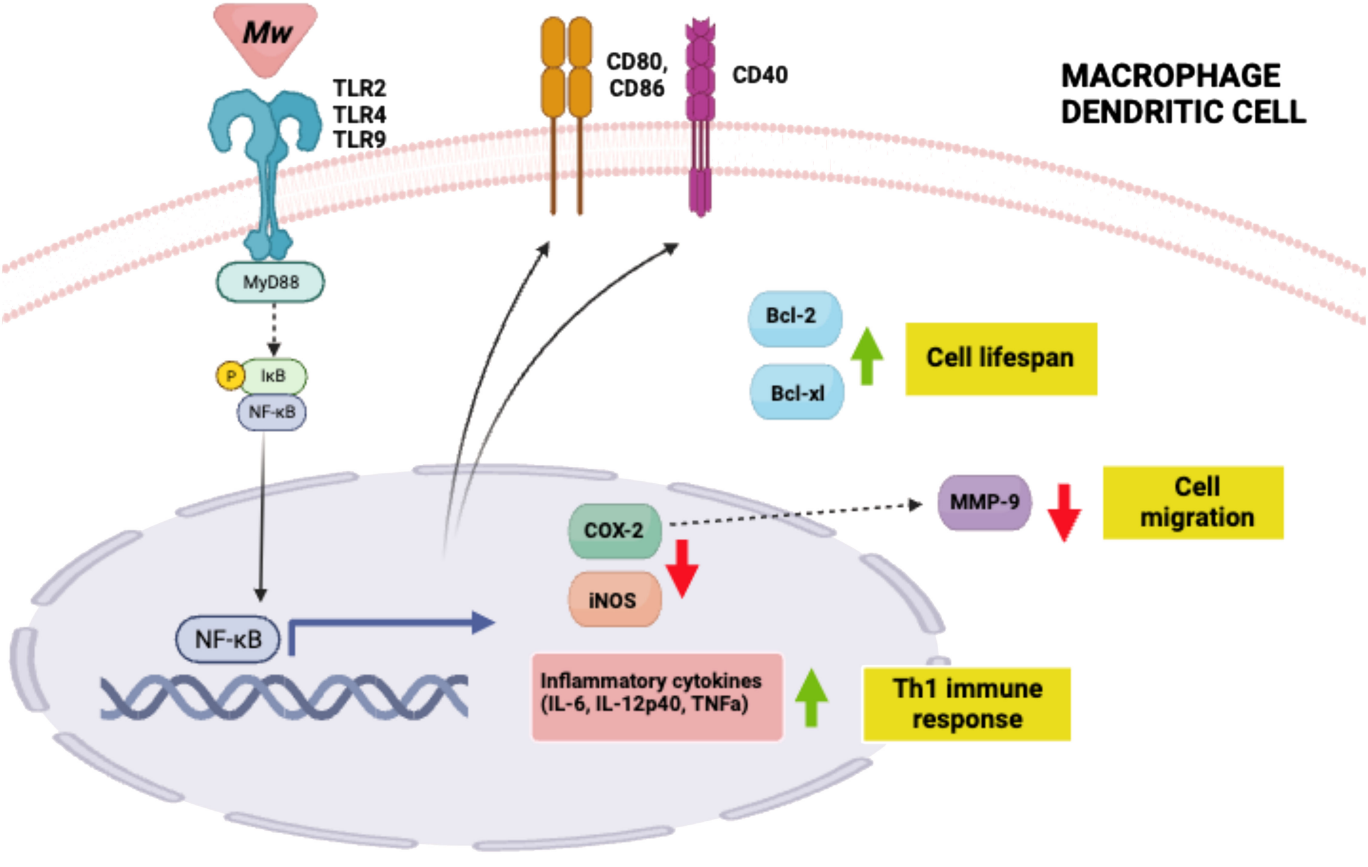
Mw mechanisms of action summary. Mw binds to TLR 2, 4 or 9 on macrophages and dendritic cells to activate NFkB.Thisinturnleadstoinflammatory cytokine production and subsequent Th1 cell polarization; indirect MMP-9 downregulation driving decreased cell migration; increased anti-apoptotic proteins which promote cell lifespan; and increase in APC co-stimulatory molecules. Created with BioRender.com.

One mechanism by which Mw carries out its immunomodulatory functions is by binding to TLR2 on antigen presenting cells (APCs) like macrophages and dendritic cells (DCs), with subsequent downstream signaling through MyD88 (27–30). Indeed, the Mw activation of APCs is lost in MyD88^-/-^ mice. (29, 30) Subsequent signaling through MyD88 facilitates active nuclear translocation of nuclear factor κB (NF-κB), resulting in transcriptional activation of pro-inflammatory cytokines IL-12p40, TNFa, IL-6, and nitric oxide in macrophages and DCs. In addition to promoting cytokine production and presentation of processed antigens, Mw also promotes the up-regulation of the co-stimulatory molecules CD40, CD69, CD80, and CD86 by immune cells (28, 31, 32). This, in turn, promotes polarization of macrophages from M2 to M1 and pushes CD4^+^ T cells toward a Th1 immune response. Both of these outcomes contribute to cell-mediated immune responses and are required for host defense against intracellular viruses, bacterial pathogens, and cancerous cells (28, 31, 32). Mw also induces expression of major histocompatibility complex class II (MHCII) molecules, which are essential for presentation of exogenous antigens to activate CD4^+^ T cells (31, 32). In addition to activating APC’s, Mw treatment also leads to enhanced APC survival through increased expression of Bcl-2 and Bcl-xl (28) (**Figure 1**).

Mw also induces T cell proliferation in a dose dependent manner, and thus is associated with an increase in CD4^+^ and CD8^+^ T cells in both peripheral blood mononuclear cells (PBMC) and in disease affected organs (23, 32–34). Mw induced changes, compared to untreated mice, in T cell frequency in the immune microenvironment include increased frequency of CD4^+^ and CD8^+^ T cell subsets include Ki67^+^CD8^+^ T cells (proliferating T cells), CD69^+^CD8^+^ T cells (activated T cells), CD44^+^CD62^+^ cells T cells (central memory T cells), as well as CD44^+^ CD62L^−^ effector memory T cells. For optimal T cell proliferation following administration of Mw, the presence of both CD4^+^ and CD8^+^ T cells is required, and T cell proliferation is reduced significantly in their absence. In addition, CD4^+^ helper T cells are required for lymphocyte proliferation after Mw vaccine administration (26, 35).

In the presence of disease, Mw administration increases infiltration of immune cells in the diseased organ, which includes an increase in immunostimulatory CD4^+^ and CD8^+^ T cells and decrease in immunosuppressive cells expressing FoxP3, PD-1, CTLA-4, IL-10, and TGF-β (31, 32, 34). In addition to T cell infiltration, Mw administration also induces a change in tumor resident immunosuppressive DCs toward Siglec-H^+^ plasmacytoid DCs (pDCs), found to contribute as much as ~80% of the tumor infiltrating DCs in a B16F10 melanoma model (32). pDC secretion of IFN-α induces proliferation of T cells and IL-12 cytokine production, as these effects are drastically reduced in IFNR1^-/-^ mice (32). Mw stimulates secretion of proinflammatory cytokines IFN-γ, IFN-α, IL-2, IL-12, TNF-α, IL-6, and nitric oxide in a dose-dependent manner, as demonstrated specifically for IL-2 and IFN-γ secretion (36). Mw has no effect on IL-4 and IL-5, but represses immunosuppressive cytokines IL-10 and TGF-β, suggesting the immune response generated following Mw administration is a Th1 type response (24, 36, 37). Further, reduction in the T regulatory cell (Treg) population following Mw administration seems to be the result of effective modulation of STAT4-STAT5 and CCL22 (36), and is supported by the reduction of Treg cytokines such as IL-10. This change in cytokine profile is seen in regardless of disease pathology, such as cancer or TB. Thus, there is a shift in the immune microenvironment from immunosuppressive to immunostimulatory after Mw administration (33–36).

Mw efficacy, as measured by the ability to change the immune microenvironment from immunosuppressive to immunostimulatory, has an inverse correlation with disease induced immunosuppression. For example, high disease induced immune suppression, as observed in large tumors, is associated with resistance to Mw (38). Mw has been evaluated for its efficacy by various routes of administration including ID, nasal, aerosol to lung, intralesional and intravenous (33–42). The ability of Mw to change the immune microenvironment improves when its administered directly to the diseased organ or in proximity to the diseased organ (intranasal route or aerosol for lung diseases) compared to parenteral (subcutaneous) administration (33, 39–43).

Mw is synergistic with therapies that reduce disease burden (e.g. chemotherapy for infection or tumor) and therapies that reduce disease induced immune suppression (e.g. GITR agonist antibody or STAT3 inhibitor) (31, 34, 37, 38, 41, 44, 45). Combining Mw with these therapies is associated with an increase in absolute number of tumor-infiltrating lymphocytes (TIL) and an increased frequency of CD4^+^ and CD8^+^ T cells with a decrease in FoxP3 and PD-1 expressing TIL (31, 34). Mw induced immune changes facilitate classical antigen presentation as well as cross presentation of antigens and the subsequent generation of antigen specific immune responses, which includes generation of antigen−specific polyfunctional T cells (IFN−γ^+^TNF−α^+^IL−2^+^ T cells or IFN−γ^+^TNF−α^+^ T cells) (33, 46).

### 2.2 Other mechanisms of action

Matrix metalloproteinase-9 (MMP-9) is an enzyme primarily involved in extracellular matrix degradation. With this function, MMP-9 plays a role in cancer cell migration and invasion and is directly involved in recruitment of macrophages to the site of infection, contributing to granuloma maturation and bacterial growth. Mw has been shown to downregulate MMP-9 in melanoma (47, 48), inhibition invasion. Further, *in vitro* Mw significantly downregulates MMP-9 and iNOS in murine peritoneal macrophages, which was shown to be mediated through cyclooxygenase-2 (COX-2). Co-incubation of LPS-treated macrophages with Mw caused a significant drop in the levels of LPS-induced COX-2 and iNOS transcripts. In addition, Mw functionally regulates COX-2 and iNOS expression not only by controlling the rate of their transcription, but also importantly, by limiting the nuclear to cytoplasmic transport of their corresponding transcripts and thus maintaining low levels of COX-2 and iNOS proteins (48). CXCR4 is a known regulator of MMP-9 (49), and Mw treatment induced a significant and dose-dependent decrease in CXCR4 expression in B16F10 melanoma cells (50); thus Mw may be altering MMP-9 through CXCR4 and/or COX-2 signaling.

There is evidence that Mw targets desmocollin-3 (DSC3) expressing cancer cells (51, 52). DSC3 is a transmembrane calcium-dependent glycoprotein belonging to the cadherin family of cell adhesion molecules found in the desmosome (intercellular junctions that provide strong adhesion between cells). DSC3 is associated with expression of p53, as a p53 mutation results in DSC3 downregulation (53, 54). Similarly, mutations in p63 and the ΔNp63α isoform result in DSC3 downregulation (55). DSC3 is also expressed in many types of cancers, including squamous NSCLC, head and neck cancer, melanoma, esophageal, colorectal, chondrosarcoma, pediatric acute lymphoblastic leukemia, and ovarian, among others (51, 52, 56– 57). DSC3 has been shown to serve as a prognostic marker for some cancers, with low expression indicating poor prognosis, suggesting it could be a novel tumor suppressor in some cancers, such as prostate (53, 58). Given this role for DSC3, the association between Mw and DSC3 is intriguing. In fact, because DSC3 is identified as a predictive biomarker for Mw efficacy as a cancer treatment, Mw has been granted orphan drug status by the FDA for the treatment of DSC3-expressing non-small cell lung cancers (59).

In a study examining the cytotoxicity attributed to Mw and different Mw fractions, only the heat killed Mw fraction showed significant cytotoxicity in several cancer cell lines, as assessed by poly-(ADP-ribose) polymerase (PARP) and DNA fragmentation assays. Additionally, a 60° C heat killing technique showed significantly greater cytotoxicity compared to an autoclave heat kill technique. Use of the PARP assay suggested that cytotoxicity could be attributed to caspase-mediated apoptosis (60). Combination of Mw with chemotherapy, like cisplatin, is synergistic for cytotoxic effects and involves intrinsic apoptosis (Caspase-9, Apaf-1 expression) and inactivation of NFκB (61).

## 3 Areas of therapeutic use

### 3.1 Leprosy

Leprosy is a chronic infectious disease caused by the organism *M. leprae*. Those who develop clinical signs of leprosy present with characteristics of leprosy clinically described as indeterminate (I), polar tuberculoid (TT), borderline tuberculoid (BT), borderline (BB), borderline lepromatous (BL), or polar lepromatous (LL) (4, 62). Patient immune status usually dictates clinical spectrum of disease (4).

Preclinical studies indicating the utility of Mw in management of leprosy have been confirmed by human clinical studies. Initially, sera from leprosy patients were found to react with Mw antigens (63), suggesting cross-reactivity. In early clinical trials for leprosy, ID administration of Mw was found to convert lepromin negative healthy contacts of leprosy patients and leprosy patients to stable lepromin positive (a surrogate for therapeutic efficacy) (15–18). Further, the skin reaction to Mw was found to be proportionate to the dose (64). These results led to the development of Mw for both prophylaxis and treatment of leprosy. Mw was evaluated for its prophylactic efficacy in 28,948 healthy household contacts of multibacillary patients belonging to 272 villages in Ghatampur, Kanpur (India). All were randomized to receive two doses of Mw or placebo. The first dose of Mw had 1 x 10 (11) heat-killed bacilli (Mw), and the second dose had half of the initial dose of bacilli and was given 6 months after the first dose. The contacts were followed up at 3, 6, and 9 years after the initial vaccination for identification of disease cases and any side-effects caused by vaccination. Protective efficacy of the Mw vaccine was 68.6%, 59%, and 39.3% at the end of 3, 6, and 9 years, respectively, after administration (21). For evaluation of therapeutic efficacy of Mw, in a single arm study of leprosy patients receiving the standard therapy of a multidrug therapy (MDT) consisting of rifampin, clofazimine, and dapsone, were also administered Mw every 3 months. The beneficial effects of Mw immunotherapy were evident from the marked clinical improvement and a more rapid bacterial clearance compared to MDT alone (65–72). Further, the addition of Mw was associated with immunological recovery and histological upgrading (4, 16, 73–76). The findings described above from single arm studies have subsequently been confirmed by controlled clinical trials (16, 72, 77–80). In these trials, compared to the control arm, Mw administration resulted in faster bacterial clearance and granuloma clearance. Indeed, bacterial clearance leading to cure (bacterial negativity) was achieved around 40% faster compared to the control group. Interestingly, based on the response to Mw, patients can be identified as either fast or slow responders. HLA antigens HLA-A28 and DQw3 (DQw8 + 9) were found to be associated with slow responsiveness, while DQw1 and DQw7 were found to be associated with more rapid responsiveness to Mw (81). Further, the Mw vaccine was well tolerated, inducing minimal side effects. At 24-48 hours post-vaccination, there was a local reaction to the vaccination in the form of erythema and induration, and at 4 weeks post-vaccination, nodule formation. In some cases, there was ulceration of the nodule, which healed on its own in few days. Finally, addition of Mw was associated with decrease in type II reaction (Erythema nodosum leprosum) (79).

In studies evaluating the roles of Bacillus Calmette Guerin (BCG) and Mw vaccine in untreated leprosy patients with a high bacterial index, the BCG vaccine was marginally better, defined by faster bacterial clearance and clinical improvements, compared to Mw; however, these two vaccines were equally effective at histopathological improvement (80).

### 3.2 Tuberculosis

Tuberculosis (TB) is a contagious infectious disease caused by *M. tuberculosis*, which is estimated to infect a quarter of the world’s population and this, is the leading cause of death from a single infectious agent (82). Unfortunately, the current vaccine, BCG, has limited efficacy, therefore a new treatment strategy is urgently needed. The antigenic similarities between TB and Mw suggest Mw vaccine has potential as a treatment for TB (19–21), and initial studies in animal models support the promise of Mw vaccination for preventing *M. tuberculosis* infection (24, 26, 33, 39, 83, 84). Early studies indicated that following exposure to the Mw vaccine, T cells in mice predominately upregulated IL-2 and IFNg, and this response outperformed an inactivated *M. tuberculosis* vaccine (24). The cytokine profile initiated by Mw vaccine suggested that Mw induced immunity was conferred primarily by Th1 cells (24). It was subsequently found that *in vivo* depletion of CD4^+^ and CD8^+^ T cells impaired the ability of Mw to confer resistance to TB in mice, as assessed by colony formation, cytokine production, and *in vivo* disease (26).

In guinea pig models of TB, Mw immunized animals had improved pulmonary pathology and reduced bacterial load in several organs in comparison to BCG immunization. This improved protection was most likely due to a notable granuloma-specific immune response and early increase in IL-12, TNFa, and IFNg, indicating an amplified protective Th1 response (39), similar to Mw induced immunity in mouse models. Additionally, in the guinea pig model, administration of Mw by an aerosol route is critical for induction of a local lung response. Further, in guinea pig models of TB, Mw upregulates CXCL10 and CXCL12, cytokines known to be key for their activity against TB in both prophylactic and therapeutic settings (85, 86). In addition, when the lungs of infected animals were evaluated, Mw treated animals were found to have higher numbers of activated APCs and lymphocytes and displayed markers of a protective Th1 immune response earlier in the course of disease (41). Overall, the Mw vaccine appears to provide a more durable protection against TB as compared to the BCG vaccine (39, 40).

In addition to Mw therapy alone, results from animal studies demonstrated faster bacterial clearance when Mw was given in addition to chemotherapy compared to chemotherapy alone (39–41, 85–88). In comparison to chemotherapy alone, the combination of Mw and chemotherapy restored the pro/anti-inflammatory cytokine balance, as indicated by an upregulation in pro-inflammatory cytokines, such as IFN-γ, IL-2, IL-12, and TNF-α, and the suppression of anti-inflammatory cytokines, such as IL-10 and TGF-β (37). When Mw was combined with chemotherapy in a multidrug resistant-TB (MDR-TB) mouse model, there was an observed reduction in the overall bacterial load in the lungs, as well as a suppression in the number of colony forming units in both the lung and spleen (87).

*M. tuberculosis* suppresses host autophagy responses in macrophages, which results in improved bacterial survival and resistance to therapy. Thus, another mechanism that might provide protection against TB is effects on macrophage autophagy. Indeed, Mw vaccination is a potent inducer of autophagy in macrophages by its ability to promote phagosome maturation and phagolysosome fusion, which enhances clearance of *M. tuberculosis* from the macrophages (88).

#### 3.2.1 Role of the route of administration on protective efficacy of Mw against tuberculosis

Mw delivered by nasal route or as an aerosol to the lung resulted in improved protection in comparison to a subcutaneous injection route (33, 39–41). It appears that the nasal/aerosol inoculation stimulates the recruitment of activated CD4^+^ and CD8^+^ T cells to the lung, engaging a stronger memory T-cell response as compared to other vaccination routes (33). Mechanistically, the CXCR3-CXCL11 regulatory axis is primarily responsible for T cell recruitment to the lung (33).

#### 3.2.2 Human clinical studies

Mw has been evaluated for its safety and efficacy in patients with pulmonary TB in clinical studies. In randomized clinical trials of patients with active pulmonary TB, the combination of standard of care chemotherapy and Mw treatment reduced the time to sputum conversion by half as compared to chemotherapy treatment alone (89, 90). Additionally, the use of Mw in combination with chemotherapy substantially improved the cure rate in patients with pulmonary TB, with upward of 97.96% patients cured (45). Thus, addition of Mw every 2 weeks to standard of care chemotherapy leads to faster sputum conversion than chemotherapy alone (45, 89, 90).

Category II pulmonary TB (Cat II PTB) includes patients who have failed previous TB treatments or have relapsed or defaulted during previous treatment. Failure to treat Cat II PTB patients effectively leads to increased prevalence of MDR-TB, and an effective treatment of Cat II PTB would help in containing the infection before conversion to MDR-TB. Mw was evaluated in a large multi-center randomized clinical trial for immunotherapeutic potential as an adjuvant for standard anti-tubercular treatment (ATT). In the study, 1022 patients diagnosed with CAT II PTB were randomized 1:1 to receive either ATT alone or Mw along with ATT. Sputum smear and culture examinations were measured at multiple time points. Addition of Mw significantly decreased *M. tuberculosis* bacilli detectable in culture at all time points beginning 4 weeks after initiation of treatment. Sputum culture conversion was 67.1% vs 57% for Mw plus ATT and ATT alone, respectively (P=0.0002, OR=1.86, 95% confidence interval (CI): 1.31–2.64) at 4 weeks, and this trend continued at each subsequent visit. The cure rate was 94.2% and 89.17% for Mw plus ATT and ATT alone, respectively (P=0.02, OR=1.97, 95% CI: 1.06–3.75), at the end of the 39^th^ week. The difference between the two arms was significantly higher in a subgroup of patients for whom ATT had a lower cure rate, for example patients with resistance to one drug (100% for Mw plus ATT vs 81.6% for ATT alone; P = 0.028), or patients with a highly bacillary load (3+) (93% for Mw plus ATT vs 84.4% for ATT alone; P = 0.035) (45).

##### 3.2.2.1 Tuberculin conversion

The immunogenic potential of Mw to provide protection against TB was evaluated in patients in 50 tuberculin negative HIV positive patients suffering from active pulmonary TB. All patients were found to be anergic to tuberculin protein and were given 0.1 ml Mw by ID route over their deltoid. When patients were revaluated 3 months after administration of Mw, 48 (96%) had tuberculin conversion and had become tuberculin positive despite being HIV positive (90).

##### 3.2.2.2 TB vaccine

Mw was administered to 28,948 healthy household contacts of leprosy patients belonging to 272 villages in Ghatampur, Kanpur (India). All were randomized to receive two doses of Mw or placebo. The first dose of 1x10^9^ Mw was followed 6 months later with a second dose of 5x10^8^ Mw. All were evaluated 10-13 years later for incidence of TB in the interim. In the placebo group, 1.11% had TB compared to 0.70% (P < 0.01) in the Mw group. The protection provided by Mw vaccine was synergistic with BCG, as only 0.53% had TB after receiving Mw along with BCG scars (91).

##### 3.2.2.3 Tubercular pericarditis

Tuberculous pericarditis (TBP) is a severe manifestation of TB disease. Elimination of *M. tuberculosis* bacilli is a key requirement for TBP treatment. The overall survival rate following a standard four drug regimen is around 26%. (92, 93) Corticosteroids are the only non-invasive form of treatment available to reduce symptoms caused by excessive inflammation in TBP. In a prospective randomized study (2-by-2 factorial design) the effects of adjunctive glucocorticoid therapy and Mw immunotherapy in patients with TBP were evaluated in 1400 adults with definite or probable TBP (IMPI trial) (46, 92, 93). There was no significant difference in the primary outcome (composite of death, cardiac tamponade requiring pericardiocentesis, or constrictive pericarditis) between patients who received prednisolone and those who received placebo (23.8% and 24.5%, respectively; hazard ratio, 0.95; 95% CI: 0.77-1.18; P = 0.66) or between those who received Mw immunotherapy and those who received placebo (25.0% and 24.3%, respectively; hazard ratio, 1.03; 95% CI: 0.82-1.29; P = 0.81) (93). Patient samples from the IMPI trial were examined for the efficacy of Mw combined with prednisolone therapy and were characterized for the influence of Mw and prednisolone on *Mycobacteria* specific CD4^+^ and CD8^+^ T cell responses at 2, 6, and 24 weeks after treatment initiation. Neither prednisolone nor Mw therapy alone resulted in significantly modulated purified protein derivative (PPD)-specific Th1 CD4^+^ T cell responses throughout the 24-week period compared to the placebo group. However, combination treatment resulted in increased *Mycobacterium*-specific CD4^+^ T cells. After 24 weeks of combination therapy, the greatest increase in frequency was seen in IFNγ+IL-2^+^TNF^+^CD4^+^ T cells, IL-2^+^TNF^+^CD4^+^ T cells, and TNF^+^CD4^+^ T cells. However, an important limitation of this study was that T cell responses were only assessed in peripheral blood, while the most robust responses would have likely been at the disease site (46).

### 3.3 Warts

Warts are the result of infection of the epidermis with human papilloma virus (HPV). Intralesional immunotherapy using mumps, measles, and trichophyton skin test antigens is safe and effective for the treatment of HPV infection when administered by ID or intralesional route, as demonstrated in a few large case series and controlled studies (94). Mw has been evaluated for its efficacy against cutaneous and anogenital warts following ID or intralesional administration.

#### 3.3.1 Intradermal Mw in cutaneous wart

In a double-blind, randomized, controlled study, the efficacy of 0.1 ml ID Mw or PPD administered every 2 weeks for a total of six doses was compared in 64 patients with multiple viral warts (≥ 5 warts). Mw was found to be better at reducing the number of warts (50.4% PPD vs 80.6% Mw), reducing the wart size (53.7% PPD vs 70.5% Mw), inducing a complete response (50% PPD vs 69% Mw), and increasing the speed of reduction (3^rd^ vs 4^th^ follow-up onwards). However, in the Mw group, an injection site reaction was also more frequent with 34.4% developing an ulcer, of which 12.5% had a discharge. These events were managed with tablet doxycycline (100 mg, twice daily) + tablet ofloxacin (200 mg, twice daily) + tablet azithromycin (500 mg, once daily) until the ulcer healed (95).

In a controlled clinical trial, 36 patients with warts were randomized to receive PPD, Mw, or Measles, Mumps, Rubella (MMR) ID every 2 weeks. Though there was no difference in overall efficacy, resolution was faster in the groups receiving Mw or MMR compared to PPD. In this case, the downregulation of the Th2 cytokine IL-10 and upregulation of the Th1 cytokines IL-1 and IFN-γ were attributed wart clearance (96).

#### 3.3.2 Intralesional Mw in cutaneous warts

In a four-arm randomized controlled trial, the efficacy and safety of intralesional injection of 0.1 ml Mw, 0.5 ml MMR vaccine, 0.2 ml vitamin D3, or 0.5 ml saline (placebo) repeated every 2 weeks were compared in 200 patients with ≥ 2 extragenital cutaneous warts. Complete response was seen in 66%, 58%, 55%, and 64% in the injected wart and in 64%, 52%, 53%, and 62% in a distant wart, in vitamin D3, Mw, MMR, and placebo, respectively (p > 0.05). The Mw group took a lesser mean time (7.1 ± 3.1 weeks) for complete clearance of warts. Pain, erythema, and swelling at the injection site were the most common side effect in all study groups, with vitamin D3 inducing significantly more side effects (52%). Fever was noted only after the 1st follow-up visits in two patients in the Mw group and one patient each in the vitamin D3 and placebo groups (97).

In a prospective randomized controlled clinical trial in patients with > 2 warts either Mw (0.1ml) or MMR (0.5 ml) was administered intralesional at the base of wart (single largest for Mw and 2–3 largest warts for MMR). The injections were repeated at the same site after 3 weeks for an additional three injections or until complete clearance of all the warts. Both treatments were found to be identical in clearance of warts, although the reduction in wart size was faster in patients receiving Mw. Pain at the injection site was more frequent in the MMR arm (63.34% for MMR vs 3.33% for Mw) while erythema/inflammation at the injection site was more frequent in the Mw arm (63.34% for Mw vs 13.3% for MMR) (97, 98).

In a single arm study, 40 patients with ≥ 3 warts received 0.2 ml Mw ID, followed by an intralesional injection into three to five warts after 2 weeks. Mw injection was repeated every week until either complete clearance of warts or a maximum of 10 injections (12 weeks), whichever was achieved earlier. Complete clearance of warts was observed in 83% (33/40) of patients, with 23 patients also experiencing resolution of distant, untreated warts. Recurrence was seen in three patients during the mean post-treatment follow-up period of 4.48 months (99).

In a retrospective analysis of 44 patients treated with intralesional Mw, complete wart clearance was achieved in 24 (54.5%) patients (100). Cosmetically acceptable response to therapy (>75% clearance) was achieved in 37 (84.1%) patients. Wart response at distant sites was seen in 38 (86.3%) patients. None of the 18 patients with complete response had recurrence during the mean follow-up of 5.27 ± 1.7 months (100).

#### 3.3.3 Refractory/difficult to treat warts

In a prospective randomized study 66 patients with refractory (defined as lack of response or a partial response to at least two interventions in last 3 months) extragenital warts (≥ 1 cm in size) received either Mw or liquid nitrogen (LN). Mw was administered intralesionally in a large wart every week beginning 2 weeks after ID injection, and LN was sprayed to result in a frozen halo of 2 mm around the base of the wart and was repeated every 2 weeks. Both treatments continued until 12 weeks of treatment were received or complete clearance of the treated lesions was achieved. All patients were followed up at 4-week intervals for 16 weeks. In this study, complete cures were identical in both groups (60.6% vs 57.6%). However, clearance of distant warts was significantly higher in the Mw group (46.2% for Mw vs 8.7% for LN; P = 0.004). Side effects of pain (23.3% for Mw vs 100% for LN; P < 0.001) or erythema (73.3% for Mw vs 58.6% for LN) were less in the Mw group compared to the LN group. Fever (100°F-104°F) for 2 to 3 days during the initial 3 to 4 days of Mw injection was recorded in 43.3% (13/30) of patients, and regional lymphadenopathy following the first intralesional Mw injection was noted in 10% (3/30) of patients (101).

A single arm efficacy study of intralesional Mw was evaluated in 30 patients with warts at difficult-to-treat sites, such as palms, volar surface of fingers, plantar surface of feet, or the subungual and periungual regions. All patients received 0.1 ml of Mw in a single wart, which was repeated at intervals of 4 weeks, until complete clearance of all warts or a maximum of ten injections. Complete response of warts, both at the injected and untreated distant warts, was seen in 28 of the 30 patients (93.33%) with a mean of 43.71 days to clearance following an average dose of 0.186 ml of Mw (102).

#### 3.3.4 Intralesional Mw in anogenital warts

The efficacy of intralesional Mw or topical Imiquimod (5%) cream was compared in a double-blind randomized clinical trial of 89 patients who had one or more anogenital wart(s) that had a surface area of 10 mm^2^ or greater. Complete resolution was seen in 59% of patients in the Imiquimod group and 67% of patients in the Mw group. Side effects such as erosions or ulcerations (64% for Imiquimod vs 47% for Mw), erythema (30% for Imiquimod vs 9% for Mw; P = 0.02), burning sensation (16% for Imiquimod vs 4% for Mw; P = 0.090), were higher in the Imiquimod group, while side effects such as edema/swelling (25% for Imiquimod vs 31% for Mw), nodule (2% for Imiquimod vs 44% for Mw; P = 0.001), and fever (16% for Imiquimod vs 31% for Mw; P = 0.09) were higher in the Mw group. Absence of HPV was seen in 30 of 52 samples evaluated by PCR. Significant reduction in HPV11 was seen only after Mw treatment (103).

In an open label study, patients with multiple and/or giant anogenital warts were treated with Mw. In this study, 0.1 ml Mw was initially administered ID in both deltoids. Two weeks later, intralesional injection into the genital warts was administered every week until either complete clearance or a maximum of ten injections (69). Warts cleared completely in eight out of nine patients (88.9%). In the remaining non-responsive patient, a giant perianal wart was reduced to less than 5% of its volume after 10 intralesional injections. Adverse reactions were noted in four patients, which were all reversible. No recurrence was seen in patients after a mean follow-up of 5.1 months. The treatment was well tolerated (104). Overall, intralesional immunotherapy of warts with Mw seems to be a promising approach, as it leads to resolution of both treated and distant, non-treated warts.

### 3.4 Sepsis

Sepsis is a syndrome caused by a dysregulated host immune response to infection (105). Almost 49 million incident cases and 11 million deaths occur worldwide annually because of sepsis (106). The ICU mortality varies from 12% to 40%, whereas hospital mortality can be as high as 47% (107). Traditionally, sepsis was believed to be a condition of uncontrolled inflammation resulting from an unrelenting innate immune response and concomitant suppression of adaptive immunity. However, current evidence suggests that both proinflammatory and anti-inflammatory responses occur simultaneously early in the course of sepsis due to simultaneous activation of both innate and adaptive immune mechanisms (108, 109). Recent evidence suggests that the sepsis continuum includes an immune paralytic state, which may play a significant role in sepsis (108). Because of the complexity of the immune response, many therapies targeting only a single inflammatory pathway have failed. In contrast, evidence suggests that immunomodulatory agents, such as Mw, can overcome the immunosuppressive state induced by sepsis (28). Mw has been evaluated in human clinical studies where it was administered by ID as well as intravenous route (109–114).

#### 3.4.1 Intradermal administration of Mw for sepsis

In a prospective randomized, double-blind placebo controlled study, Mw efficacy in combination with standard therapy was evaluated in 50 patients with severe, presumed gram negative sepsis (96). All patients were randomized within 48 hours of first organ dysfunction to receive either 0.3 ml Mw or saline ID over deltoid for 3 consecutive days and then observed for 28 days for all-cause mortality (96). Patients receiving Mw had a significant reduction in the number of days on mechanical ventilation (median, 6 days and 9 days for Mw and saline, respectively; P = 0.025), the number of days in ICU (7 days and 12 days for Mw and saline, respectively; P = 0.006), length of hospital stay (10 days and 16 days for Mw and saline, respectively; P = 0.007), delta sequential organ failure assessment (SOFA) score (P = 0.027), and secondary bacterial infection (5% vs. 14%; P = 0.009). Across groups, there was no significant difference in mortality, and overall, Mw was well tolerated (111).

A large randomized, double-blind, placebo-controlled, parallel-group study (N=202) was conducted in the ICUs of five tertiary care centers in India to evaluate the efficacy and safety of Mw in critically ill patients suffering from presumed gram-negative sepsis and requiring intensive care. The use of Mw was associated with a significant reduction in mortality in these patients; 9/101 patients died in the Mw arm compared to 20/101 patients in the control arm (estimate difference, 0.11; 95% CI: 0.01-0.21; P = 0.04). After adjusting for culture status, baseline SOFA score, age, and sex, the odds of reduction in mortality were 0.37 (95% CI: 0.15-0.9) in patients that received Mw (110).

A meta−analysis of the two randomized controlled trials discussed above, including a total of 252 sepsis patients, revealed a 43% lower mortality in patients receiving Mw compared to control (RR: 0.57; 95%CI: 0.33–1). There was a standard mean difference in vasopressor days (0.38; 95% CI: -1.20–0.44), length of ICU stay (0.46; 95%CI: −1.44–0.51), and delta SOFA score (0.88; 95%CI: −1.66 to − 0.10) that all favor Mw treatment. Similarly, there was a reduction in relative risk of secondary infection (0.75; 95%CI: 0.19–3.01) and ventilator associated pneumonia (0.6; 95%CI: 0.28–1.56), also favoring Mw treatment (112).

#### 3.4.2 Intravenous administration of Mw for sepsis

Because ID administration of Mw is challenging in ICU settings, the intravenous route of administration has also been explored for effects on sepsis outcomes.

The safety and efficacy of intravenous Mw as an adjuvant to standard treatment in 30 gram-negative sepsis patients was evaluated (113). All patients received 0.3 mL of Mw diluted in 100 mL normal saline, which was given as a slow intravenous infusion over at least 15 minutes, every day for 3 consecutive days in addition to the standard-of-care therapy for sepsis (111). Generally, all vital parameters and mean SOFA score showed a significant improvement from day 2 onwards. Out of 30 patients, 17 (56.7%) required ventilator support, and death was reported in seven (25%). The mean duration of ICU stay, hospital stay, vasopressor therapy, and ventilator support were 8.9, 14.4, 5.3, and 6.1 days, respectively. No patients were reported to have a secondary infection (113).

A prospective study evaluated the efficacy and safety of intravenous Mw in 20 patients with gram−negative sepsis. All patients received 0.3 ml of Mw diluted in 100 ml normal saline that was administered as a slow intravenous infusion over at least 15 minutes, along with standard of care (114). Comparing patients who survived to those who died in this study, a significant difference in the baseline median SOFA score was reported (8 vs 4; P < 0.01). Further, a change in SOFA score from baseline to day 7 and to day 14 correlated significantly with Mw administration. On day 4, the mean total leukocyte count showed a significant change from baseline after Mw administration. In this study, none of the patients had mortality due to side effects of the drug. Hence, the adjunctive Mw via an intravenous route is safe in patients with severe sepsis (114).

### 3.5 COVID-19

#### 3.5.1 Mw administration for prevention of COVID-19 infection (prophylaxis)

Due to its role as a modulator of innate immune responses, Mw vaccine was evaluated for its ability to prevent COVID-19 prior to the availability of SARS-CoV-2 specific vaccines and was found to be useful in preventing the development of symptomatic COVID-19. For example, in a cohort of 3831 healthy subjects, ID Mw provided protection against the development of symptomatic COVID-19 (Hazard ratio (HR) = 0.297; 95% CI: 0.209-0.422; P <0.0001) and COVID-19 specific hospitalization (HR = 0.144; 95% CI: 0.078-0.268, P <0.0001). This translates to a protective efficacy (1 - HR) of 70.3% (95% CI: 57.8-9.1). Protection was slightly, but not significantly, better in those who had received two injections of 0.1 ml Mw over each deltoid compared those who received one injection (odds ratio = 0.7474; 95% CI: 0.4237-1.3181; P = 0.3145). The protective effect of Mw appear to last for at least 6 months (115). A self-limiting local injection site reaction that persisted for more than 2 weeks was seen in 26.92% subjects that received Mw (115).

In a controlled study, Mw was administered to 50 health care workers as a prophylaxis against COVID-19. The control arm was made up of another 50 health care workers. In this study, the protective efficacy of Mw was found to be 82.35% (95% CI: 50%-96%) with a HR of 0.15 (95% CI: 0.04-0.5, P = 0.003). The management of acquired COVID-19 infection required hospitalization in 35.3% (6 of 17) of patients in the control group but no patients in the Mw group (P = 0.01). All three COVID-19 infections in the Mw group occurred 21 weeks following Mw administration. Ulceration at the local injection site was observed in 8% of the subjects, which healed with scar formation, as also seen following BCG vaccine administration. Only one subject had mild fever (37.5°C) for less than 2 hours, 24 hours after administration, which was self-limiting (116). Mechanistic study of subjects revealed that an increased level of NKG2C, a marker of activated NK cells, was associated with COVID-19 protection, and that Mw increased NKG2C levels (116). When the Covid vaccine (ChAdOx1) became available, all 50 health care workers that received Mw prior to vaccination were compared with 200 others who received the ChAdOx1 vaccine only. The symptomatic COVID-19 infection rate was significantly lower in the Mw + ChAdOx1 group compared to the ChAdOx1 only group (4% vs 17.9%, respectively; P = 0.01). Thus, prior Mw administration augmented the protective efficacy of ChAdOx1 vaccine (HR = -0.46; P = 0.009). Protection against COVID-19 was associated with increased frequency of NKG2C^+^ cells in both groups (117).

#### 3.5.2 Mw administration for treatment of critically ill COVID-19 patients

Multiple studies examined the effect of Mw on critically ill patients infected with SARS-CoV-2. In these studies, Mw was compared against the best standard treatment at the time. Mw, given at a dose of 0.3 mL daily for 3 days, resulted in a significant reduction in the length of stay of patients in the intensive care unit and mortality in patients with severe disease. Mw was found to improve clinical parameters, including normalization of lung architecture (computed tomography scan), a significant increase in oxygen saturation, and a reduction in/weaning off ventilation. Furthermore, there was no evidence of any adverse effects caused by Mw during the hospitalization of COVID-19 patients; although, local Injection site reactions were described (118,). The concomitant improvement in biological and clinical parameters offers indirect evidence of the beneficial/effective role of Mw for COVID-19 patients (119–121). In a separate study, when Mw was administered intravenously at a dose of 0.6 ml for 3 days, it was also associated with improved lung function and no side effects (122).

### 3.6 HIV

#### 3.6.1 Effect on CD4 count

The human immunodeficiency virus (HIV) is associated with decreased immunity, specifically a depletion of CD4^+^ T cells. In a clinical trial testing Mw alone or in combination with antiretroviral therapy, patients were evaluated to determine the effect of Mw on CD4^+^ T cell count in HIV positive patients. CD4^+^ T counts were increased by 108.96% compared to baseline in patients receiving both Mw and antiretroviral therapy, suggesting a synergistic action. However, a significant increase (80.22% compared to baseline) was also seen in patients receiving Mw alone, suggesting that Mw was an effective immune response enhancer (123).

#### 3.6.2 Tuberculin conversion

Patients with HIV are not protected against TB (tuberculin negative), and these patients have increased incidence of TB in comparison to non-HIV infected patients. Given this incidence of TB, Mw vaccine was evaluated in 50 tuberculin negative HIV patients. Of these 50 participants, 48 had tuberculin conversion (a delayed hypersensitivity reaction, indicating the status of cellular immunity against TB) after 90 days, which indicates the ability of Mw vaccine to provide cell mediated protective immune response against TB (90).

### 3.7 Steroid resistant optic neuritis

There are currently no established treatment protocols for corticosteroid refractory optic neuritis. Six patients with documented idiopathic unilateral optic neuritis who did not improve with methylprednisolone followed by oral steroids, as per the Optic Neuritis Treatment Trial (ONTT), were administered 5 mL Mw in 500 mL normal saline, 30 days after their last of dose of steroids had been administered. This treatment was repeated after 3 months. Patients were monitored for change in the best-corrected visual acuity (BCVA), pupillary reaction, color vision, visual field (VF) examination (when possible), fundus examination and photography, visually evoked potential (VEP) testing BCVA, pupillary reaction, and color vision. All patients that received Mw showed improvement in visual acuity, color vision, and pupillary reaction. Visual field monitoring was possible in four patients; all four had a centrocecal scotoma that persisted post-steroid therapy but resolved 1-month post-Mw therapy. Three patients with disc edema had resolution of disc edema. In these six patients, no adverse events were seen and there was no recurrence of disease up to the 5-year follow-up (124, 125).

### 3.8 Plaque psoriasis

The effect of Mw was evaluated in 24 patients with plaque psoriasis. The study group patients received 0.1 mL of Mw ID. This was followed by a booster dose (0.1 mL) after 3 weeks. The patients were evaluated after 1 and 4 months based on reduction of their Psoriasis Area and Severity Index (PASI) score, a widely used measurement in psoriasis trials that assesses and grades the severity of psoriatic lesions and the patient’s response to treatment. In the present study, at the end of 4 months, 16.6% of patients showed marked improvement, and 62.5% of patients showed moderate improvement. Thus, this trial revealed the potential of Mw as an adjuvant in the therapy of plaque psoriasis (126). However, another study using a dosing strategy that provided patients with a total of four doses had contrasting results (127). In this trial, fewer than 40% of patients showed only mild to moderate improvement, which also only lasted a short amount of time (127).

### 3.9 Leishmaniasis

*Leishmania donovani* is a parasite that can cause three associated syndromes, cutaneous, mucocutaneous, and visceral leishmaniasis, and is endemic predominately in Europe, Northern Africa, the Middle East, Asia, and part of South America (128). *L. donovani* primarily infects macrophages and DCs and through these cells *L. donovani* acts to suppress the immune system of the host (129). The realization that leishmania shared common antigens with both *M. leprae* and *M. tuberculosis* (130) raised the potential for Mw to have a therapeutic effect against this infection as well.

When investigated as a monotherapy or in combination with low levels of the anti-leishmania drug, Amphotericin B (AmpB), it was observed that both treatment modalities upregulated Toll-like receptor 4 (TLR-4) signaling in infected macrophages (131). Additionally, both treatment approaches were effective in stimulating a pro-inflammatory response and restored inducible nitric oxide synthase (iNOS) expression, an indicator of proinflammatory macrophages, which might allow the cells to clear the parasite (130). In a related article, Mw administered alone or with AmpB prevented infection by activating macrophages, and in the case of an active infection, Mw induced nitric oxide production, restored the Th1 response, and enhanced parasitic clearance by macrophages (132). Further, this data indicated that IL-12 played a critical role in mediating the protection afforded by Mw and AmpB treatments by showing that suppressing IL-12 signaling in mice negated the protective effect of all treatments evaluated (132). It was subsequently identified that Mw treatment of infected macrophages enhanced the expression of dual-specificity phosphatase 6 (DUSP6) and downregulated DUSP1 through activating TLR-4 signaling, which enhanced IL-12, p38 phosphorylation, and iNOS expression (133). In a drug resistant model of leishmaniasis, the use of Mw as an adjuvant combined with heat-induced promastigotes, or the flagellated form of *L. donovani*, was effective in controlling infection (134). This combination effectively stimulated a Th1 and Th17 response, which depended upon active TLR-2 signaling from DCs (134). However, it should be noted that there is some inconsistent evidence supporting the use of Mw in the treatment of *Leishmania* infection. Work by Tandon et al. suggested that Mw was an ineffective treatment for managing acute and chronic *L. donovani* infections in mouse and hamster models (135). These inconsistent findings highlight the need for further evaluation of Mw in the context of *Leishmania* treatment.

## 4 Mw use as a vaccine adjuvant for therapeutic and prophylactic vaccine

Adjuvants are incorporated into vaccine formulations to enhance, accelerate, and prolong antigen-specific immune responses. Alum, which induces a strong humoral immune response, has been used as an adjuvant for years and has an excellent safety profile (136). However, there is a need for an adjuvant that promotes Th1 immune responses and has the same safety profile as Alum. Due to its Th1 immune-stimulating properties, Mw has been studied for its adjuvant properties. To test for adjuvanticity, different fractions of Mw were evaluated in comparison to whole Mw for immunostimulatory or immunoadjuvant activity. In these experiments, the whole cell wall fraction induced a significant Th1 immune response, while the cell wall skeleton induced a strong Th2 immune response (137).

Mw has also been evaluated as a vaccine adjuvant for increasing antibody titer and cell-mediated immune responses in mouse models for the management of cancer, as well as for the prevention of pregnancy and infections (138–146). Clinical trials have suggested that anti-hCG vaccination can be applicable for both anti-fertility and anti-cancer. One study investigated the inclusion of Mw with the anti-hCG vaccine. Elevated antibody titer, T cell recall proliferative and cytokine responses to hCG were observed in the Mw adjuvant treatment group. Additionally, Mw increased vaccine immunogenicity in mice of diverse genetic backgrounds (including in traditionally low-responder murine strains), leading to enhanced titers of bioneutralizing anti-hCG antibodies in sera which exhibited cytotoxicity toward tumor cells (141). The addition of Mw as an adjuvant to an alum-containing vaccine increased IgG1 as well as IgG2a and IgG2b antibodies, which suggests both Th1 and Th2 responses (138).

Survivin, a tumor antigen, is overexpressed in many different cancers and is not expressed in normal tissue (147). Downregulation of the expression or function of survivin inhibits tumor growth and increases spontaneous apoptosis in different tumor models (148). Thus, the *in vivo* efficacy of recombinant survivin as a cancer vaccine was tested along with the adjuvants alum and Mw in the breast cancer model 4T1, which expresses high levels of survivin compared to normal breast tissue (149). Immunization with the combination of this antigen (survivin) and adjuvant (alum and Mw) was immunogenic and significantly suppressed tumor growth in mice (139). Combination of 20 µg recombinant survivin and Mw was also effective in suppressing growth of 4T1 tumors (P = 0.04) (140). However, it had no effect on metastasis to the lung. Combining survivin with luteinizing hormone-releasing hormone only improved antitumor effect marginally, but did prevent metastasis to the lung (140).

A rabies DNA vaccine from the pgp.LAMP-1 construct (glycoprotein G gene with Lysosomal Associated Membrane Protein-1 targeting sequence) conferred partial protection (60%) against intracerebral challenge with 20 LD_50_ of the rabies challenge virus standard (CVS) strain in BALB/c mice. To improve vaccine efficacy, several adjuvants were investigated to supplement the vaccine, including Mw. Sera analyzed for antibody titer from immunized mice showed that Mw elicited an IgG titer with an IgG1/IgG2a ratio <1, indicating an enhancement of cell-mediated immunity. Further, cytokine analyses showed an enhanced Th1 response, with higher IFNg levels compared to the other adjuvants tested. Although Mw supplementation led to 70% survival post intracerebral viral challenge, Alum supplementation, which induced a Th2 response, led to 100% survival (143).

Mw has also been evaluated as an adjuvant with recombinant H5N1 proteins (HA, NP, M2e). In this study, Mw was administered twice at a 3-week interval. The addition of Mw provided complete protection against homologous as well heterologous viral challenge. Combination of HA+NP+M2e with adjuvant Mw had a better efficacy than Mw adjuvanted HA vaccine. Protective efficacy of 80% was achieved following single dose of HA+NP+M2e with adjuvant Mw when challenged by heterologous virus. This protection was associated with induction of IFN-γ, CD8^+^ T cytotoxic cells, and CD4^+^ T helper cells (144, 145).

Vaccine adjuvants are generally administered concurrently with the vaccine/antigen. However, Mw was found to boost vaccine efficacy even when administered before or after vaccine administration (40, 117). For example, in animal studies, Mw boosted BCG vaccine efficacy when given following BCG vaccination (40). In humans, Mw improved protective efficacy of COVID-19 vaccine when administered before vaccination (117). Mw has also been evaluated for efficacy with recombinant or inactivated chikungunya virus vaccine along with other adjuvants. In this case, Mw was inferior in its adjuvant activity compared to Alum and liposomal adjuvants (146).

As a vaccine adjuvant, Mw can be used to improve efficacy of prophylactic vaccines to provide faster, better, and more sustained protective antibody titers and therapeutic vaccines for better cell mediated immune responses. Mw administration can also be considered to improve protective efficacy of subsequent vaccination, which may be particularly helpful in infants.

### 4.1 Cancer

#### 4.1.1 Non-small cell lung cancer

Initially two separate studies were undertaken to evaluate safety and efficacy of Mw when used along with chemoradiotherapy or chemotherapy in non-small cell lung cancer (NSCLC) (150, 151). These studies were followed by a larger study wherein Mw was evaluated for improved efficacy when added to chemotherapy. Notably, DSC3 expression, which may be a marker of Mw efficacy, is known to be expressed in squamous NSCLC and is used to for differentiating squamous NSCLC from adenocarcinoma of lung (151, 152).

#### 4.1.2 Mw with chemoradiotherapy

Whether addition of Mw to chemoradiotherapy increased chemoradiotherapy efficacy was evaluated in a controlled study in which 20 NSCLC patients were randomized in a 1:1 ratio to receive chemoradiotherapy alone (control arm) or in combination with Mw. Compared to the control arm, addition of Mw was associated with an improvement in quality of life, as measured by Karnofsky performance status, and tumor regression. Two-thirds of patients receiving chemoradiotherapy with Mw showed regression in tumor size (P < 0.01), which was significantly better than in patients receiving chemoradiotherapy alone. Tumor regression also reflected an improvement in lung function (p<0.001) (151). Patients receiving Mw also tolerated therapy well, with no patients discontinuing therapy (p < 0.05). Finally, responses seen in patients receiving chemoradiotherapy with Mw were durable and no progression was seen during the follow up period.

#### 4.1.3 Mw with chemotherapy

The addition of Mw to chemotherapy (Platinum doublet) was evaluated in a controlled clinical trial in which randomized patients with stage IIIB or IV NSCLC were assigned in a 1:1 ratio to receive chemotherapy alone or chemotherapy plus Mw. Mw (0.1ml) was administered every 2 weeks ID during chemotherapy. Addition of Mw led to significant improvement in tumor regression (the response rate was 38.5% and 27% for combination and chemotherapy alone, respectively), median survival (11 months vs 7 months), and 1-year survival (46.2% vs 36.4%). Mw was well tolerated, and no additional systemic side effects were seen. Thus, addition of Mw to chemotherapy provided longer survival without any additional toxic side effects compared to the patient group who received chemotherapy alone (151).

The efficacy of adding Mw to chemotherapy was further evaluated in a multicenter, open label, controlled clinical trial of 221 treatment naïve adult subjects with advanced (stage IV and/or IIIb) NSCLC. The patients were randomized in a 1:1 ratio to receive chemotherapy alone (four cycles of cisplatin-paclitaxel, n=112) or chemotherapy with additional Mw (n=109) (49). The study was conducted under IND #13176 approved by the U.S. FDA in addition to the Indian regulatory agency. Similar to the previous studies, there was an increase in response rate by 10% (47% vs 37%) in the group receiving chemotherapy plus Mw compared to the group receiving chemotherapy alone. This was associated with a 17.48% increase in survival rate by (33.89% vs 16.41%) at the end of 1 year. Subgroup analysis revealed a significantly increased benefit in median survival for patients having squamous NSCLC in intent to treat analysis (HR = 0.55; 95% CI: 0.32-0.95; P = 0.046), and in those who received all four cycles of planned chemotherapy (HR =0.40; 95% CI, 0.17-0.96; P=0.04) (51). The side effect profile was identical in both groups and no additional systemic side effects due to Mw were observed. The benefit seen in squamous NSCLC may be due to the presence of DSC3 in squamous NSCLC and the absence of DSC3 in non-squamous NSCLC before the initiation of chemotherapy (152, 153). This has led to orphan drug designation for Mw by U.S. FDA for DSC3-expressing NSCLC (59).

### 4.2 Melanoma

To test the efficacy of Mw either prophylactically or as a therapeutic against melanoma, a syngeneic mouse model of melanoma, B16F10, was chosen because this is a very aggressive tumor model and poorly immunogenic. In this model, Mw was tested for immunotherapeutic efficacy using two different treatment regimens. In the therapeutic regimen, Mw was given after implantation of melanoma cells. In the prophylactic + therapeutic regimen, one Mw injection was given before tumor implantation and the rest were given after implantation of melanoma cells. Decrease in tumor growth was observed in both treatment groups as compared to control, but in the latter group, tumor growth was delayed, and tumor volume was significantly less than control. In the group receiving Mw both prophylactically and therapeutically, there was no tumor growth in about 40–50% of mice, which contributed to the prolonged survival of the treated mice as compared to control. To more deeply understand the Mw mediated immune activation, the tumor microenvironment (TME), tumor draining lymph node, and spleen were analyzed. Induction of a Th1 response and higher infiltration of immune cells in the TME was observed in all Mw treated mice compared to control. A large fraction of these immune cells was in an activated state, as confirmed by phenotypic and functional analyses. Interestingly, the percentage of Treg cells in the TME of treated mice was reduced compared to control. Additionally, Mw in combination with chemotherapy was also evaluated in the B16F10 model, which induced a better response as compared to chemotherapy alone (31). Further, evaluation of the efficacy of Mw at different doses (3 × 10^6^, 5 × 10^6^, or 7 × 10^6^ bacilli/mice) in the B16F10 model revealed dose-dependent Mw effects. In addition, Mw treated groups (5 ×10^6^ and 7 ×10^6^ bacilli/mice) significantly reduced cancer associated cachexia as compared to untreated mice. Cachexia index further influenced the survival of mice directly, as the Mw treated group had approximately one third of the total number of mice alive until day 60 compared to day 45 in the control group (50).

Metastatic melanoma is characterized by abnormal proliferation of melanocytes. Invasion and metastasis are the main reasons for the high mortality rates associated with melanoma, and neither radiation therapy nor chemotherapy are effective treatments. Therefore, a therapeutic strategy that aims to control invasion and metastasis through the development of effective anti-invasive agents will most likely improve outcomes. Matrix metalloproteinases (MMPs) have frequently been reported to facilitate tumor invasion and metastasis (154), with MMP-9 acting as one of the most important mediators of tumor migration and invasion (155). Interestingly, Mw downregulated MMP-9 expression in the highly invasive B16F10 melanoma model, which was sensitive to Mw treatment. Mw suppressed MMP-9 gene expression through blocking the activation of NF-κB and AP-1 transcription factors via PKCα-mediated PI3K/AKT and ERK-1/2 signaling, therefore reducing invasion and metastasis of B16F10 cells (47). Thus, Mw inhibited MMP-9 expression and subsequently reduced the invasiveness of B16F10 melanoma cancer. Peroxisome proliferator-activated receptor γ (PPARγ) has a known inhibitory role against NF-κB/p65 (156), thus Mw was investigated for its effects on PPARγ expression in B16F10 tumor cells. Mw treatment resulted in a significant increase in PPARγ, an increase that was equivalent to the known PPARγ agonist Rosiglitazone. Additionally, there was a synergistic effect when Mw was combined with Rosiglitazone for PPARγ expression, tumor growth, and MMP-9 expression (50).

Glycyrrhizic acid, a bioactive compound purified from the plant *Glycyrrhiza glabra* has been reported to exhibit immunomodulatory, antimicrobial, and antitumor activities (157). One study examined Glycyrrhizic acid and Mw combination therapy. Although Mw alone showed little response to tumor volume, Mw combined with Glycyrrhizic acid showed a significant synergistic effect in reducing advanced stage melanoma tumor volume (44).

Another possible mechanism for Mw efficacy against metastatic melanoma, as observed in the B16F10 mouse model, may be through the TLR2-MyD88 signaling axis (29). It’s possible through this pathway, that Mw was able to redirect tumor-associated macrophages (TAM) from M2 to M1 *in vitro*. However, in advanced-stage B16F10 melanoma, Mw was unable to generate an effective anti-tumor response *in vivo*. In this case, Mw treated tumors accumulated Tregs, which neutralized the anti-tumor immune response via TGF-B and IL-10 dependent ERK-1/2 MAPK and STAT3 activation in TAM. However, when Mw was combined with a GITR agonist antibody, which can suppress Tregs, advanced stage tumors regressed. In this case, Mw plus anti-GITR combination therapy complemented one another’s function *in vivo* to generate an efficient host anti-tumor immune response (38).

Since peritumoral administration of Mw is not always feasible in clinical settings, a novel delivery system was developed. Chakraborty, et al. designed a chitosan nanoparticle-based delivery system to target Mw adjuvants inside the TME (158). Chitosan alone also has anti-cancer activity, which includes inducing tumor cell apoptosis (159). The Mw nanoparticles (NP) were intravenously administered on day 3 following tumor implantation into a B16F10 melanoma mouse model. Following administration, a maximum concentration (C*max*) of Mw NP was observed in the plasma after 1 hour, and by 6 hours post-administration, C*max* was reached inside the tumor. Mw NP immunized mice exhibited reduced tumor volumes and significantly prolonged survival compared to untreated control mice. The immune cell profile of immunized mice had a significantly higher number of activated T cells, as indicated by the increased frequency of CD44^+^CD4^+^ and CD44^+^CD8^+^ T cells, which have a crucial role in suppressing tumor growth, as compared to the untreated controls. Further, Mw NP treated tumors had an increased frequency of F4/80^+^ macrophages, which expressed activation markers like MHC-II, CD86, and CD40, suggesting M1 polarized TAMS that have a high anti-tumor activity. Interestingly, Mw NP not only increased the frequency of CD11c^+^ DCs within the TME but also significantly upregulated the expression of activation markers on their surface, further sculpting the immune response toward Th1 type. Supporting this, *ex vivo* stimulation of splenocytes isolated after Mw NP treatment resulted in secretion of Th1 cytokines INFg, TNFa, and IL-12 (158). Additionally, in a preliminary study, when mice from different treatment groups that did not develop tumors were rechallenged with tumor cells after 3 months from the final therapeutic dose, mice from the Mw NP group did not develop any tumors, suggesting a long-lasting protective memory response generated by Mw therapy.

Mw given as a monotherapy has been evaluated in 12 patients with advanced refractory/recurrent melanoma in a single arm study approved by US FDA. All patients received Mw twice a week for 4 weeks and once a week for another 4 weeks. This regimen was repeated in patients whose tumor did not progress. Of the 12 patients, response to Mw was seen in three patients. In these patients, the response was durable with improved survival (160). Administration of Mw was associated with reduction in circulating Treg (FoxP3+) cells.

### 4.3 Head and neck cancer

The use of Mw has been examined in head and neck cancer either as a monotherapy or concomitant with standard of care (chemoradiotherapy, chemotherapy). Multiple different regimens of Mw have been evaluated, and the best results were seen when Mw (0.2 ml) administration was initiated 2 weeks prior chemoradiotherapy (161).

#### 4.3.1 Mw with chemoradiotherapy

In a prospective, randomized, single center, controlled study, 50 patients with locally advanced (stage III, IVa, IVb) head and neck squamous cell carcinomas (HNSCC) were randomized to receive chemoradiotherapy alone or in combination with Mw. In the combination group, Mw (0.2ml, ID) was administered every week starting 2 weeks prior to treatment. In this trial, complete responses were more frequent in patients that received Mw [80% (20/25) vs 32% (8/25); p=0.003). No disease progression was seen in those receiving Mw.^170,.^ Addition of Mw was also associated with reduced toxicity, as grade IV toxicity oral mucositis (n=3), hematological toxicities, (n=8) and skin toxicities (n=3) were seen only in the group that received chemoradiotherapy alone (161).

In a prospective clinical trial, 60 patients with locally advanced head and neck cancer (LAHNC) planned to have chemoradiotherapy (64 Gy/32 fractions over 6.2 weeks plus intravenous cisplatin 40 mg/m2 weekly for six doses; CCRT arm) were randomized to receive chemoradiotherapy alone or with ID Mw (0.2 ml) weekly for six doses (MwCCRT). The complete response rate was observed to be 70% and 63.3% (p =0.752), no evidence of disease (NED) at the 6 month follow up was seen in 63.3% and 53.3% in MwCCRT and CCRT arms (P = 0.612), respectively. Grade 2 skin and mucosal toxicity were observed in 40% and 83.3% (P = 0.033) in MwCCRT and CCRT arms, respectively. This study suggests that the use of weekly Mw vaccine plus weekly cisplatin is safe, and MwCCRT may be superior to CCRT in terms of a marginally better response rate and higher locoregional control but with significantly reduced toxicities (162, 163).

Ninety one patients with advanced head and neck cancer were randomized in 2:1 ratio to either receive 0.1 ml Mw ID every week for 6 to 10 weeks with chemoradiotherapy (n=61) or chemoradiotherapy alone (164). In this trial, complete responses were more frequent in patients receiving Mw plus chemoradiotherapy (8.1%, n=5) compared to chemoradiotherapy alone (3.33%, n=1). The response rate was identical in both groups. (93.4% vs 93.3%) (164). Grade III skin reaction and mucositis were seen in 16.7% (5/30) of patients receiving chemoradiotherapy only. Overall, patients receiving Mw plus chemoradiotherapy had a lower incidence of hematological side effects [14.4% (9) vs 33.33% (3)], gastrointestinal side effects [4.19% (3) vs 66.66% (4)], grade II skin reactions [16.4% (14) vs 50% (15)], and grade II mucositis [21.3% (13) Vs 46.6% (14)]. A local injection site reaction in the form of small local abscess was seen in 15% of patients that received Mw (164). Patients receiving Mw also had improvement in Karnofsky performance Scale (quality of life) by a mean of 10 compared to the group receiving chemoradiotherapy alone (164).

#### 4.3.2 Mw as a monotherapy

In a prospective single-arm study of patients’ ineligible for any other therapy, injection with 0.1 mL Mw ID every week for 8 weeks was administered as a monotherapy. Partial response was seen in 27/75 patients. Healing of ulcer/fistula was seen in 4/5 patients, dysphagia improvement was seen in 7/15 patients, and improvement in voice was seen in 5/19 patients (165).

In a prospective single arm study, 100 terminally ill symptomatic head and neck cancer patients who had exhausted their therapeutic options and were ineligible for any further treatment were enrolled to receive 0.1 mL Mw ID every week for 4 weeks followed by 0.1 mL Mw every month for 4 months. In this study, an improvement in quality of life and symptoms was seen in 82/100 patients and pain relief was reported in in 27/100 patients. Control of other symptoms was also observed in 15% patients. Patients with dysphagia, ulcer, and fistula benefitted the most. The effect lasted for 3-6 months (166). Of these patients, 5% developed constitutional symptoms and had injection site reactions (166).

#### 4.3.3 Mw with paclitaxel

In a retrospective analysis of 28 patients with relapsed/refractory head and neck cancer, Mw and 80 mg/m^2^ paclitaxel were administered every week. All patients had received at least one previous chemotherapy treatment with 17 receiving two and six receiving more than two chemotherapy treatments. Previous chemotherapy received included platinum (27), taxane (13), triple metronomic (5), 5-fluorouracil (3), and gemcitabine (4). At a median follow-up of 3.4 months (range, 0.2-18.1), six patients had stable disease and 20 patients (71.4%) had progressed on treatment. The median progression-free survival and overall survival were 2.9 months (95% CI, 2.36-3.48) and 4.9 months (95% CI, 3.78-5.99), respectively. The combination therapy was well tolerated (167).

### 4.4 Bladder cancer

Mw has been evaluated in management of non-muscle invasive bladder cancer (NMIBC) as a monotherapy and along with radiotherapy in advanced bladder cancer due to its Th1 response enhancing ability (168–170).

#### 4.4.1 Mw monotherapy in treatment naïve non muscle invasive bladder cancer

In a multi-centric randomized controlled clinical trial 122 patients with NMIBC were randomized in a ratio of 1:1 to receive either ID Mw or intravesical BCG following complete transurethral resection (TUR). Mw (0.2 ml total; 0.1 ml over both deltoids) was administered following TUR. Subsequently, 0.1 ml was administered every 2 weeks for first 3 months, then monthly for 6 months, and thereafter once every 2 months for 6 months. Patients in the BCG arm had BCG injections once a week for 6 weeks then maintenance therapy was administered each week for 3 consecutive weeks at 3, 6, 12, 18, and 24 months. All patients were followed until recurrence or 37 months, whichever was earlier. Recurrence free survival was identical in both groups (168).

#### 4.4.2 Mw as a monotherapy in BCG recurrent or refractory non muscle invasive bladder cancer

In an open label, single arm study 22 patients with either BCG unresponsive or recurrent NMIBC with ECOG performance status scores of 0 or 1 were recruited. All patients received 0.1 ml of Mw over both deltoids on day -1 (total of 0.2 ml) and 0.1 ml every 2 weeks for the first 3 months, every month for the subsequent 6 months, and once every 2 months for the next 6 months (168). The study protocol was approved by the FDA, Drug Controller General of India, and Institutional Ethics Committees of all participating study centers. Of the 20 evaluable patients, 35.0% (7/20) were recurrence free for more than 30 months and at the last follow-up. In six patients without recurrence at the last follow up, DSC3 expression was evaluated in pretreatment biopsy samples. Recurrence free survival beyond 30 months was seen 2.8 times more often in DSC3 expressing patients, which might suggest role for DSC3 as a predictive biomarker (169).

#### 4.4.3 MW along with radiotherapy in advanced bladder cancer

ID Mw has been evaluated in 6 patients with muscle invasive bladder cancer with hematuria (with T3 tumor staging), who were not willing or unsuitable for cystectomy and undergoing external beam radiation therapy. The tumor size ranged from 2 × 3 cms to 8.9 x 7 cms. All patients became asymptomatic within 6 weeks and achieved complete response with no recurrence of disease (170).

### 4.5 Breast cancer

In a mouse model of highly metastatic breast cancer (4T1), Mw was tested alone or in combination with the cytotoxic drug cisplatin and/or the chemo-sensitizing drug 1′-S-1′-acetoxychavicol acetate (ACA). The Mw treatment alone did not induce significant tumor regression. However, the triple combination therapy of Mw/ACA/cisplatin was able to elicit a significantly stronger response compared to placebo control and cause the highest tumor volume reduction. The efficacy of Mw given with cisplatin/ACA combination therapy is thought to be due to its role in targeting the host immune system. As such, the combination of Mw, as an immune potentiator, with ACA and cisplatin, as cytotoxic agents, resulted in a synergistic effect against the tumors (171, 172).

### 4.6 Pancreatic cancer

Mw effects on pancreatic cancer have been evaluated *in vitro* and *in vivo.* Mw demonstrated dose-dependent inhibition of growth of the MIA-Pa-Ca pancreatic cell line *in vitro* (173). In a murine pancreatic ductal adenocarcinoma (PDAC) tumor model, Mw treatment and Mw plus gemcitabine combination treatment inhibited tumor progression. Analyses of PBMCs and tumors revealed that Mw treatment was associated with an increase in CD4^+^ and CD8^+^ T cells in both PBMC and tumor infiltrating lymphocytes along with a significant decrease in expression of the immunosuppressive markers FoxP3 and PD-1 ^353^.

### 4.7 Osteosarcoma

The efficacy and immunomodulatory effect of Mw was evaluated in osteosarcoma (OS) tumor progression in a subcutaneous K7M2 syngeneic mouse model. Treatment was initiated when tumors reached a size of 100-200 mm^3^. At that time mice were given doxorubicin, ~0.5 x 10^9^ bacilli of M ID, alone or in combination with doxorubicin. Results showed that Mw decreased tumor burden and prolonged survival. In fact, Mw alone was more effective at tumor inhibition than doxorubicin alone. In addition, dividing tumor cells were significantly reduced after the Mw plus doxorubicin combination treatment. Analysis of the TME showed Treg suppression, increased mature DC: Treg ratio, and an increased infiltration of macrophages. Further, a screen of three OS cell lines identified cells with high DSC3 expression, suggesting a possible mechanism for Mw efficacy (174).

## 5 Potential side effects

Local injection site reactions are the most common adverse event from ID or intralesional Mw administration. These are usually minor, self-limiting, and occur within 2 to 6 weeks at the injection site following ID or intralesional administration. The lesion begins as a small erythematous papule, which subsides on its own without any treatment and sequelae in most cases. In some cases, the lesion ulcerates and subsequently heals with atrophic scarring in 4 to 5 weeks. Sterile pustule formation prior to ulceration is also observed (45, 51, 64, 79, 89, 90, 97, 98, 104, 115, 116, 118, 164–166, 175). Granulomatous reaction at the injection site has also been described, which can be treated with topical steroids, oral steroids, or minocycline (176–179). For example, a local injection site granulomatous lesion was reported following intralesional administration of Mw in a patient with genital warts who was seropositive for HIV (104).

Inadvertent subcutaneous or intramuscular administration of Mw leads to severe injection site reactions in the form of painful erythematous nodules leading to large ulcers and sterile abscesses. Generalized granulomatous dermatitis secondary to Mw vaccine has been documented in patients with leprosy as well as following Mw vaccine for use in COVID-19 managemnt (177–182). The granulomatous dermatitis is generally observed 3 months after the second dose of Mw vaccine. Erythema nodosum leprosum (ENL) is the most common manifestation of type 2 lepra reaction (T2LR) due to immune complex-mediated inflammation in patients of lepromatous (LL) and borderline lepromatous (BL) leprosy. Administration of Mw is associated with reduction in ENL; however in a patient with higher bacillary load and ENL treated with MDT, thalidomide, prednisolone, and subsequent addition of Mw lead to paradoxical appearance of ENL 10 days after initial Mw vaccination and recurrence 10 to 14 days following 3 subsequent Mw administrations (183).

Few systemic side effects attributable to Mw have been described. Mw has been administered along with various other therapeutic agents like antibiotics, anti-tubercular drugs, antiretroviral, chemotherapy, and radiotherapy without any drug-drug interactions or additional systemic adverse events attributable to Mw administration (36, 41, 69, 96, 151, 153, 159). Low-to-moderate grade self-limiting fever, mostly on day 2 after the ID injection, has been observed in patients with warts and bladder cancer (100, 169, 184).

## 6 Future uses

### 6.1 Cancer

As an active immunotherapy, Mw can activate CD8^+^ T cells to target DSC3 expressing cancer cells while downregulating tumor induced immunosuppression by decreasing intratumoral FoxP3 and PD-1 expressing immune cells; thus, Mw can be effectively used for management of cancers expressing DSC3. Currently available literature indicates DSC3 expression by squamous NSCLC, ovarian cancer, cervical cancer, melanoma, colorectal cancer, meningioma, esophageal squamous cell carcinoma, sarcoma, and pediatric acute lymphoblastic leukemia (51, 52, 56–58, 185–188). In these cancers, Mw can be used alone or along with other effective therapies like chemotherapy, targeted therapy, or other immunotherapies, such as checkpoint inhibitors, to improve outcome (31, 34, 37, 38, 41, 44, 45, 174). In DSC3 negative cancers, there are effective therapies described to convert DSC3 negative tumors to DSC3 positive status, even at a subtherapeutic dose (189). This phenomenon could be exploited for use to manage of DSC3 negative tumors with Mw along with effective therapies.

### 6.2 Pain relief

The downregulation of TLR4 by Mw may have beneficial consequences for neuropathic pain (personal communication—Cadila Pharma Pvt. Ltd.). Studies suggest that continuous activation or dysregulation of TLR4 signaling may contribute to various painful conditions, including neuropathic pain (61). Interestingly, the analgesic effects of opioids are secondary to their TLR4 antagonism, and TLR4 signaling leads to the undesirable effects of opioids, in addition to opioid tolerance, hyperalgesia, and allodynia (190–192). Through TLR signaling, Mw may be used to effectively reduce pain without the addiction liability associated with use of opioid analgesics. Additionally, bone pain associated with metastatic bone lesions is often due to increased osteoclastic activity. As a result of the Th1 polarizing ability of Mw and subsequent p38 down regulation, there is improvement of osteoblastic activity and a decrease in osteoclastic activity with resulting pain reduction (52, 193, 194).

### 6.3 Autoimmune disease

Mw is known to downregulate overexpressed endosomal TLRs (TLR3, 7, 8, 9) (personal communication—Cadila Pharma Pvt. Ltd.). Endosomal TLRs are overexpressed in various autoimmune diseases. Thus, use of Mw may be helpful in keeping autoimmune diseases under control by establishing immune homeostasis rather than the immunosuppression induced by current therapies like glucocorticoids and other imunomudulators (195–198). Type I diabetes is an autoimmune disease mediated by endosomal TLRs; Mw may be useful in delaying progress of type I diabetes (199, 200).

## 7 Concluding remarks

The Mycobacterium w (Mw), a heat-killed suspension derived from a non-pathogenic mycobacterium, has been widely studied as an immunomodulating agent. This review summarizes applications of Mw as a therapeutic agent for various diseases. The mechanism of action of Mw is yet to be fully understood; however, the results so far provide strong motivation to continue to develop Mw as a therapeutic for human diseases.
